# Programming the tumor microenvironment through microbiome-driven mechanisms

**DOI:** 10.3389/fcimb.2026.1857151

**Published:** 2026-06-09

**Authors:** Jhommara Bautista, Sebastián Calderón-Cevallos, Ana María Salvador-Baquero, Xavier Naranjo-Castillo, Andrés López-Cortés

**Affiliations:** Cancer Research Group (CRG), Faculty of Medicine, Universidad de Las Américas, Quito, Ecuador

**Keywords:** clinical translation, drug metabolism, immune checkpoint activity, metabolites, microbiome-derived signals, tumor microenvironment

## Abstract

The tumor microenvironment (TME) comprises interacting immune, stromal, and metabolic compartments that determine tumor behavior and treatment response. Microbial communities modulate host signaling within the TME through metabolite-driven and receptor-mediated mechanisms. Lipopolysaccharides (LPS), short-chain fatty acids (SCFAs), bile acids, and amino acid–derived metabolites engage host receptors, including Toll-like receptors, G protein–coupled receptors, and aryl hydrocarbon receptor pathways, to regulate immune cell differentiation, antigen presentation, and metabolic adaptation. These microbiome-derived signals promote context-dependent immune suppression or immune activation according to metabolite concentration, receptor engagement, and immune cell composition, thereby influencing tumor progression and immune evasion. Host-driven inflammation and metabolic constraints reshape microbial composition and function within tumor-associated niches. Microbiome-associated mechanisms contribute to tumor initiation, progression, and therapeutic response through modulation of immune checkpoint activity and drug metabolism. Major limitations include reliance on associative human data, methodological variability across sequencing approaches, contamination in low-biomass samples, and incomplete integration of multi-omics datasets. Clinical translation requires mechanistic validation, longitudinal study designs, and standardized analytical frameworks to define reproducible microbiome-associated signatures.

## Introduction

The tumor microenvironment (TME) consists of immune populations, stromal cells, extracellular matrix components, and soluble mediators that regulate tumor behavior. Immune subsets, including cytotoxic and exhausted T cells, tumor-associated macrophages, dendritic cells, and myeloid-derived suppressor cells, drive effector or suppressive immune states according to local signaling conditions. Stromal architecture and extracellular matrix composition regulate tissue architecture, immune cell trafficking, and vascular accessibility, while metabolic constraints such as hypoxia, lactate accumulation, and nutrient competition impair immune cell function and promote tumor progression through metabolic reprogramming and immune suppression (Duan et al., 2025; Lin et al., 2026).

Microbial communities have emerged as potential modulators within the TME, modulating immune infiltration, signaling, and tumor cell behavior at both local and systemic levels. Microorganisms originating from the gut or residing within tumor tissues have been identified across multiple cancer types, where localization occurs within cancer cells, immune populations, or extracellular niches. Composition varies according to tumor type and correlates with immune infiltration patterns, tumor progression, and therapeutic response, indicating tumor-specific ecological niches and microbial heterogeneity (Tingting et al., 2025; Zhang et al., 2025c; Lin et al., 2025a).

Microbiome-associated alterations in cancer involve functional alterations beyond taxonomic composition. Dysbiosis promotes chronic inflammation, genotoxic stress, immune evasion, and metabolic rewiring, while selected microbial communities support immune surveillance and tumor control depending on context. Microbiome-associated effects depend on local immune states, microbial activity, and metabolic conditions, resulting in tumor-promoting or tumor-restraining processes across different malignancies (Zou and Green, 2023; Liu et al., 2025).

Current limitations arise from reliance on taxonomic descriptions that do not resolve functional mechanisms. Associations between microbial composition and tumor phenotypes often lack mechanistic definition and causal inference. Interpretation remains constrained by variability across sequencing approaches, contamination in low-biomass tumor samples, and insufficient integration of metabolomic, spatial, and functional datasets, limiting translation into clinically actionable models (Jain et al., 2021; Chen et al., 2026).

Although most mechanistic evidence linking the microbiome to tumor microenvironment regulation derives from bacterial communities within the gut microbiome, additional microbial compartments have also been associated with cancer biology. Emerging evidence indicates that the virome and mycobiome contribute to inflammatory signaling, immune modulation, epithelial barrier disruption, and therapeutic response across multiple malignancies. However, fungal- and virus-associated interactions within the tumor microenvironment remain comparatively less characterized and lack the level of mechanistic and analytical standardization currently available for bacterial microbiome research. Accordingly, the present review focuses primarily on bacteria-derived metabolites, signaling pathways, and immune mechanisms involved in tumor microenvironment programming (Lin et al., 2026, Lin et al., 2025a).

Microbial metabolites mediate signaling between microbial activity and host pathways within the TME. Short-chain fatty acids (SCFAs), bile acids, tryptophan-derived metabolites, polyamines, and lipopolysaccharides (LPS) regulate immune cell differentiation, cytokine production, antigen presentation, and tumor cell behavior through defined signaling pathways. Evidence from developmental microbiome research supports that microbial metabolites act as systemic signaling molecules capable of influencing immune maturation, metabolic programming, and host regulatory pathways beyond compositional variation. Engagement of host receptors, including G protein-coupled receptors and aryl hydrocarbon receptor pathways, together with epigenetic regulation through histone deacetylase inhibition, modulates T-cell differentiation and effector function, macrophage polarization, immune exhaustion, and immunotherapeutic responsiveness (Chigozie and Esimone, 2026; Hu et al., 2025b).

Reciprocal interactions further define microbiome–TME dynamics. Tumor-associated inflammation, altered nutrient availability, and metabolic reprogramming reshape microbial composition and metabolite production, reinforcing feedback loops that sustain immune suppression, chronic inflammation, and tumor progression. In parallel, specific microbial configurations and metabolite profiles enhance antitumor immunity and improve response to immunotherapy, resulting in context-dependent functional effects (Bautista et al., 2026d; Chiodelli et al., 2026).

Existing reviews on microbiome–cancer interactions have largely focused on taxonomic associations, immunotherapy response, microbial metabolites, or tumor-specific microbial signatures. Many reviews examine isolated mechanisms or single cancer contexts, limiting integration of microbial-derived signals into a unified framework of tumor microenvironment organization. Interactions between microbial metabolites, host receptor sensing, immune remodeling, stromal adaptation, and metabolic reprogramming are frequently discussed as independent processes despite functional overlap within the tumor microenvironment. In addition, gut-derived and intratumoral microbial activity are commonly examined separately, although both contribute to local and systemic regulation of tumor-associated signaling networks (Situ et al., 2025; Zhang et al., 2025c). To address this gap, microbiome–tumor interactions are organized here as interconnected mechanisms involved in tumor microenvironment programming across immune, stromal, metabolic, and therapeutic dimensions.

Support for microbiome-mediated tumor regulation remains uneven across cancer types. Mechanisms involving inflammatory signaling, microbial metabolites, immune remodeling, and therapeutic response have been consistently reported in colorectal cancer (CRC), melanoma, hepatocellular carcinoma (HCC), and gastric cancer. However, many host–microbiome interactions originate from tumor-specific experimental systems and lack validation across multiple malignancies. Variability in epithelial architecture, immune composition, microbial spatial distribution, and metabolic conditions generates distinct tumor-associated ecological environments that shape microbial activity and host signaling. Experimental findings discussed in this review are therefore interpreted according to the cancer type and biological context in which they were originally described (Chen et al., 2026; Patra et al., 2025). The evidence presented throughout the manuscript is also interpreted according to the type of supporting data available, including observational cohort studies, mechanistic *in vitro* systems, preclinical animal models, and early-phase clinical intervention studies.

An additional unresolved issue involves defining how microbial-derived signals integrate into tumor microenvironment organization across malignancies. Although associations between microbiome composition and tumor phenotypes continue to accumulate, mechanistic integration linking microbial metabolites, host receptor signaling, immune remodeling, stromal adaptation, and therapeutic response remains incomplete. Resolving this limitation requires moving beyond descriptive microbiome profiling toward functionally resolved models capable of connecting microbial activity with biologically relevant tumor phenotypes. Microbiome-driven programming is therefore defined as the process through which gut-derived and tumor-resident microbial communities generate molecular mediators sensed by host pathways, leading to alterations in immune, stromal, metabolic, and therapeutic response states within the TME. Microbiome–TME interactions can consequently be interpreted as interconnected biological sequences rather than isolated taxonomic associations. For example, gut-derived SCFAs act as systemic mediators that engage G protein–coupled receptors and histone deacetylase–dependent pathways, influencing T cell differentiation and immune activity. By contrast, intratumoral bacteria generate localized signals that act directly on cancer, immune, or stromal cells, affecting antigen presentation, innate immune activation, stromal remodeling, and immune evasion. The proposed framework incorporates five interconnected components: microbial source, microbial mediator, host receptor or sensing pathway, affected TME compartment, and tumor-related outcome. Based on this structure, gut-derived and intratumoral microbial signals are examined in relation to immune remodeling, stromal adaptation, metabolic reprogramming, tumor progression, and therapeutic responsiveness within the tumor microenvironment.

## Microbiome-driven programming of the tumor microenvironment

**Figure 1:**
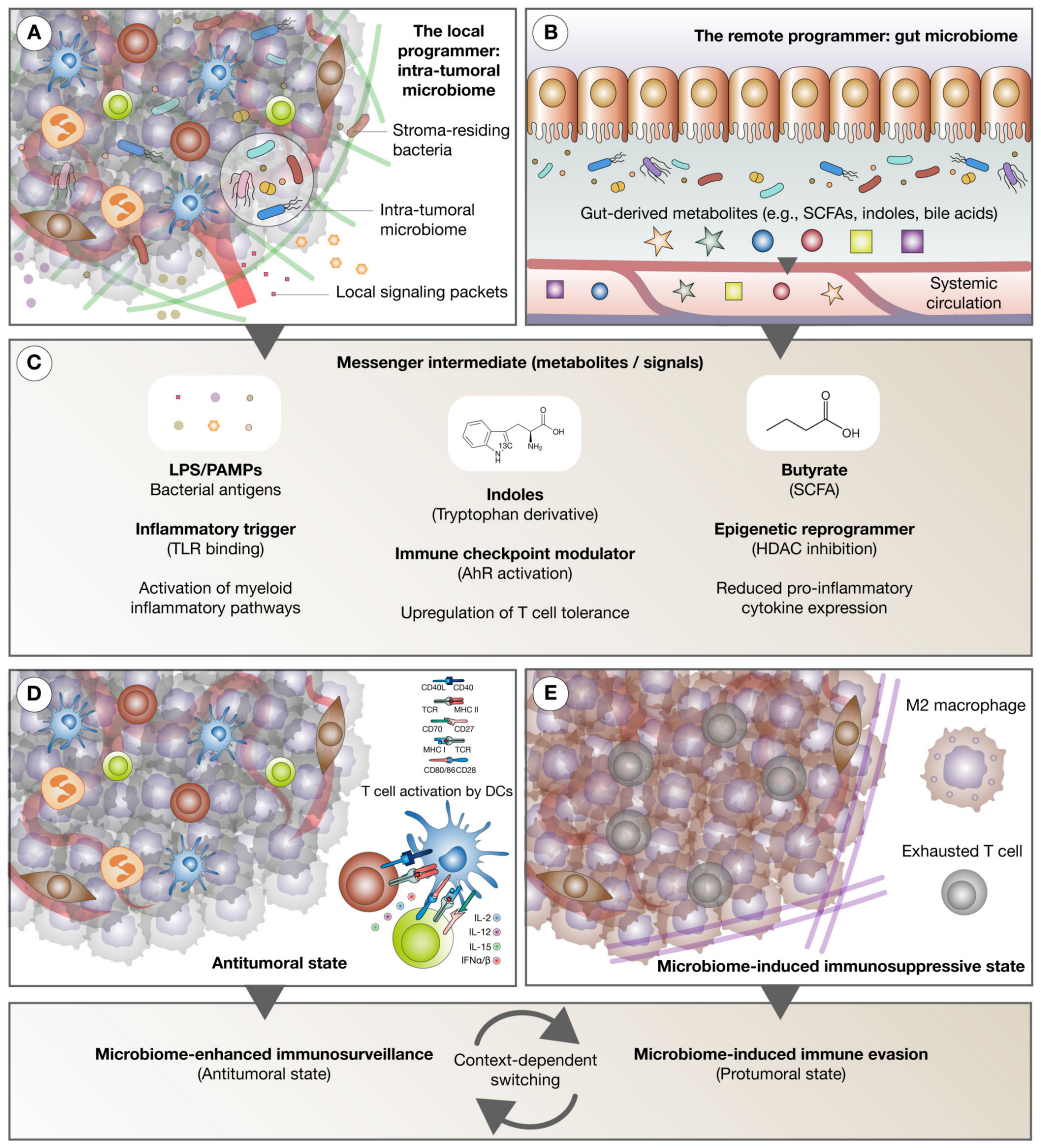
Remote and local microbiome-derived signals shape antitumoral or protumoral immune states in cancer. **(A)** The intratumoral microbiome acts as a local programmer of the tumor microenvironment, comprising tumor-resident and stroma-associated bacteria that generate spatially restricted microbial cues and local signaling packets within the tumor niche. **(B)** The gut microbiome acts as a remote programmer by producing microbial products and metabolites, including short-chain fatty acids (SCFAs), indoles, and bile acids, which enter the systemic circulation and can influence distant tumor-associated immune environments. **(C)** Signals derived from both local intratumoral and remote gut microbiota converge through messenger intermediates, including LPS/PAMPs, which trigger inflammatory signaling through Toll-like receptor pathways; indoles, which modulate immune responses through AhR activation and promotion of T cell tolerance; and butyrate, a major SCFA that acts as an epigenetic reprogrammer through HDAC inhibition and reduced pro-inflammatory cytokine expression. **(D)** Under favorable conditions, microbiome-derived signals support an antitumoral state characterized by dendritic-cell-mediated T cell activation and microbiome-enhanced immunosurveillance. **(E)** Under alternative conditions, microbiome-driven signaling promotes a microbiome-induced immunosuppressive state characterized by tumor microenvironment remodeling, including accumulation of M2-like macrophages and exhausted T cells, leading to microbiome-induced immune evasion. Together, the image illustrates how remote gut-derived metabolites and local intratumoral microbial signals may shift the tumor immune landscape between microbiome-enhanced immunosurveillance and microbiome-induced immune evasion. This context-dependent switching is influenced by microbial source, metabolite concentration, signaling duration and intensity, receptor engagement, immune-cell composition, microbial localization, and the inflammatory or metabolic state of the tumor microenvironment.

Microbiome-driven programming of the TME depends on microbial metabolites that mediate signaling between microbiota and host cells. LPS, SCFAs, bile acids, and amino acids–derived metabolites regulate immune cell differentiation, inflammatory tone, and metabolic adaptation within tumor sites. These functional outputs may connect gut-derived systemic signals with tumor-associated immune landscapes, whereas intratumoral microbial signals operate through more localized mechanisms (Feng et al., 2024; Xie et al., 2025; Bautista and López-Cortés, 2026b) (**Figure 1A**).

Gut-derived and intratumoral microbiota contribute to tumor microenvironment regulation through different biological contexts and levels of evidence. Gut microbiome-associated effects are primarily linked to circulating metabolites, microbial-associated molecular patterns, systemic immune modulation, and metabolic regulation. By contrast, intratumoral microbiota interact directly with cancer cells, immune populations, and stromal compartments within tumor tissues, influencing local inflammatory signaling and immune activity. Experimental and clinical evidence supporting gut microbiome-associated mechanisms remains stronger, particularly in studies evaluating immunotherapy response and microbial metabolite signaling. In comparison, intratumoral microbiota studies remain more limited because of low microbial biomass, contamination risk, spatial heterogeneity, and reduced causal validation (Zhang et al., 2026a; Zhai et al., 2026).

LPS drives inflammatory signaling within tumor settings. Disruption of epithelial barrier integrity facilitates systemic dissemination of LPS, enabling engagement of the CD14/TLR4/MD-2 receptor complex on innate immune cells and activation of MyD88-dependent signaling cascades. Subsequent NF-κB activation sustains IL-6, TNF-α, and IL-1β production, maintaining pro-tumorigenic inflammatory signaling. Persistent activation of this pathway contributes to immune evasion, promotes tumor cell survival signaling, and supports a microenvironment permissive to tumor progression (**Table 1**) (Tong and Lou, 2025).

**Table 1:** Major microbiome-derived mediators involved in tumor microenvironment regulation, associated host signaling pathways, and corresponding levels of evidence.

Microbial mediator	Host receptor/pathway	Affected TME compartment	Cancer context	Type of evidence	Evidence strength	Reference
LPS	TLR4/NF-κB	Innate immune compartment	CRC, gastric cancer	Preclinical + observational	Moderate	(Su et al., 2003)
Butyrate	GPCRs/HDAC inhibition	CD8^+^ T cells, Tregs	CRC, melanoma	Preclinical + mechanistic	Moderate	(Chen et al., 2025c)
Secondary bile acids	FXR/TGR5/CXCL16–NKT axis	Macrophages, CD8^+^ T cells, NKT cells	HCC, CRC	Preclinical	Limited–moderate	(Ma et al., 2018)
Colibactin	DNA damage pathways	Epithelial compartment	CRC	Mechanistic + human mutational signatures	Strong	(Jans et al., 2024)
*Fusobacterium nucleatum*	TLR4/NF-κB/PD-L1	Immune suppression	CRC	Preclinical + observational	Moderate	(Yang et al., 2017)
Indole metabolites	AhR	T cells/macrophages	Pan-cancer experimental models	Preclinical	Limited	(Hezaveh et al., 2022)

Despite the predominantly protumorigenic role attributed to chronic LPS exposure, inflammatory consequences vary according to signaling intensity, timing, and tissue context. Acute TLR4 activation may transiently enhance antigen presentation and innate immune activation, whereas persistent signaling favors chronic inflammation, myeloid-cell expansion, and immune exhaustion. Contradictory findings across tumor models likely reflect differences in microbial burden, epithelial barrier integrity, and the balance between local immune activation and sustained inflammatory damage (Cavalcante et al., 2025; Page et al., 2022).

SCFAs modulate immune responses through receptor-mediated signaling and epigenetic regulation, depending on concentration and target cell type. Butyrate enhances CD8^+^ T cell persistence and promotes transcriptional programs associated with stem-like differentiation, supporting antitumor immunity within tumor-associated lymphoid compartments (Bachem et al., 2025). Concurrently, SCFAs inhibit histone deacetylases and signal through G protein–coupled receptors, leading to alterations in dendritic cell maturation, macrophage polarization, and expansion of regulatory T cell populations. Immune effects depend on metabolite concentration, target cell populations, and tissue-specific conditions, reflecting context-dependent modulation of immune activation and suppression (Jia et al., 2024; Wen et al., 2026).

Although butyrate is frequently associated with enhanced antitumor immunity, opposing effects have also been reported across tumor settings. Elevated SCFA concentrations may promote regulatory T cell expansion and suppress effector immune responses, particularly within chronically inflamed or hypoxic microenvironments. In CRC, butyrate can support epithelial homeostasis and CD8^+^ T cell persistence, whereas in other contexts prolonged exposure has been associated with T cell exhaustion, reduced dendritic-cell activation, or tolerance-associated transcriptional programs. Divergent findings likely reflect differences in metabolite concentration, duration of exposure, tissue-specific nutrient availability, microbial community structure, and the metabolic state of immune cells within the TME. Evidence therefore supports a context-dependent rather than uniformly antitumoral role for SCFAs in cancer-associated immune regulation (Xiang et al., 2026; Sun et al., 2024).

Bile acids link microbial metabolism to immune signaling within the TME. Microbial conversion of primary bile acids into secondary bile acids generates ligands for nuclear receptors such as FXR and membrane receptors including TGR5, enabling direct modulation of immune cell function. Activation of these receptors influences macrophage polarization, natural killer cell activity, and T cell differentiation. Dysregulated bile acid profiles promote chronic inflammation and immunosuppressive environments. Taurocholic acid restricts CD8^+^ T cell infiltration and impairs effector function, whereas microbial bile salt hydrolase activity restores antitumor immune responses (Tong and Lou, 2025; Yuan et al., 2025).

The functional consequences of bile acid signaling remain incompletely resolved across cancer types. Secondary bile acids have been linked to immune suppression, impaired CD8^+^ T cell infiltration, and tumor progression in hepatobiliary and gastrointestinal tumors, yet other studies report context-specific anti-inflammatory or barrier-protective effects mediated through FXR-dependent pathways. Experimental findings also differ according to bile acid composition, receptor distribution, and anatomical site, suggesting that pooled interpretation of “bile acid effects” may obscure biologically distinct activities among individual metabolites. Variability in diet, microbial enzymatic capacity, and host metabolic status further complicates interpretation across cohorts (Cong et al., 2024; Varanasi et al., 2025).

Amino acid–derived microbial metabolites link microbial metabolism to immune regulation. Tryptophan catabolites, including indole-3-propionic acid, modulate CD8^+^ T cell function through epigenetic mechanisms such as increased H3K27 acetylation at loci controlling T cell differentiation and persistence. These effects promote expansion of progenitor exhausted T cells with self-renewal capacity and enhance responsiveness to immune checkpoint blockade. Additional microbial metabolites influence antigen presentation, T cell activation, and myeloid cell polarization, depending on ligand–receptor specificity and tissue context (Feng et al., 2024; Lei et al., 2025).

Host sensing of microbial signals involves pattern recognition receptors and metabolite-responsive pathways that translate microbial inputs into coordinated cellular responses. Toll-like receptors and NOD-like receptors detect microbial-associated molecular patterns and initiate inflammatory cascades, while receptors such as FXR and TGR5 mediate responses to metabolite-derived signals. NF-κB integrates inflammatory cues with cellular survival and proliferation programs, linking microbial sensing to tumor-associated phenotypes (Tong and Lou, 2025; Jia et al., 2025).

Intratumoral microbiota provide an additional regulatory layer through spatially localized interactions with immune and stromal compartments (**Figure 1B**). Microbial communities within tumor tissues influence antigen presentation, immune cell recruitment, and activation of signaling pathways such as STING, contributing to either antitumor immunity or immunosuppressive niches depending on microbial composition. Tumor-specific microbial signatures correlate with therapeutic response and disease progression, supporting the role of spatial heterogeneity in microbiome–TME interactions (Liu et al., 2025).

Receptor-mediated signaling and metabolite-dependent immune modulation progressively alter immune, stromal, and metabolic conditions within the tumor microenvironment, linking early microbiome-driven programming to subsequent tissue remodeling.

## Microbiome-driven remodeling of the tumor microenvironment

Microbiome-driven remodeling of the tumor microenvironment refers to the structural and cellular consequences that emerge after persistent exposure to microbial-derived inflammatory and metabolic signals. Whereas microbiome-driven programming involves receptor-mediated and metabolite-dependent signaling mechanisms, remodeling reflects the resulting reorganization of immune composition, stromal architecture, extracellular matrix dynamics, and metabolic compartmentalization within tumor tissues. These alterations generate spatially heterogeneous tumor niches associated with immune suppression, invasion, and therapeutic resistance. Tumor-resident microorganisms and translocated microbial products contribute to these processes by sustaining inflammatory signaling, altering stromal activation states, and modifying metabolite availability across tumor-associated niches (Zhai et al., 2026; Wei et al., 2025; Yu et al., 2024).

### Immune compartment

Microbiome-driven remodeling of the immune compartment arises from persistent inflammatory signaling and metabolite-dependent immune modulation previously described in microbiome-driven programming. Sustained activation of NF-κB- and STAT3-associated inflammatory networks promotes accumulation of immunosuppressive myeloid populations, impaired dendritic-cell maturation, reduced antigen presentation, and decreased cytotoxic T cell activity within tumor tissues. Progressive disruption of immune-cell composition contributes to establishment of suppressive immune niches enriched in regulatory T cells and myeloid-derived suppressor cells (Chen et al., 2025b).

In CRC, enrichment of *Fusobacterium nucleatum* is associated with increased PD-L1 expression, accumulation of myeloid-derived suppressor cells, and reduced CD8^+^ T cell infiltration, supporting formation of an immunosuppressive tumor microenvironment. Comparable associations involving *Fusobacterium* enrichment and inflammatory remodeling have also been reported in gastric tumors, although mechanistic validation outside CRC remains more limited (Wu et al., 2026; Hoffmann et al., 2025a; Duizer et al., 2025).

Microbial metabolites further modulate immune cell function through receptor-dependent mechanisms. SCFAs regulate T cell differentiation through histone deacetylase inhibition and G protein–coupled receptor signaling, influencing the balance between effector and regulatory T cell populations. Bile acids activate FXR and TGR5 receptors, altering macrophage polarization and suppressing pro-inflammatory cytokine production (Li et al., 2025).

Intratumoral microbial signals affect spatial immune organization through activation of STING and related innate immune pathways, modulating interferon signaling and immune cell recruitment according to microbial composition and metabolite availability (Fan et al., 2025).

A microbiome-dependent immune landscape emerges, characterized by reduced CD8^+^ T cell infiltration, increased accumulation of regulatory T cells and myeloid-derived suppressor cells, and impaired antigen presentation, collectively promoting immune evasion within tumor tissues (**Figure 1E**). Pan-cancer analyses show that microbial diversity correlates with immune cell composition and immunotherapy outcomes, supporting a role in determining responsiveness to immune checkpoint blockade (Zhu et al., 2025).

### Stromal compartment

Experimental evidence suggests that microbiome-associated inflammatory signaling may contribute to stromal remodeling through effects on extracellular matrix organization, fibroblast activation, and cytokine-mediated signaling. Microbial-associated cytokines and inflammatory mediators activate stromal cells and induce matrix-remodeling programs (Hu et al., 2025a). IL-6 and TNF-α derived from microbiome-activated immune cells engage STAT3 and NF-κB signaling in cancer-associated fibroblasts, leading to transcriptional upregulation of matrix metalloproteinases (MMP2, MMP9). Increased MMP activity induces extracellular matrix degradation, reduces structural integrity, and facilitates tumor cell invasion into surrounding tissues (Zhang et al., 2026b). Mechanistic evidence linking *Fusobacterium nucleatum* to extracellular matrix remodeling is strongest in CRC, where TLR4/NF-κB activation induces MMP9 expression and promotes degradation of stromal barriers associated with tumor invasion. Whether similar stromal remodeling programs occur across other tumor types remains insufficiently defined *(*Risoen et al., 2025*)*.

Microbial components further modulate adhesion and cytoskeletal organization through interactions with host cell receptors. Bacterial ligands binding to integrins and E-cadherin activate focal adhesion kinase (FAK) and downstream PI3K–AKT signaling, promoting loss of epithelial polarity, cytoskeletal reorganization, and increased cellular motility, which contribute to invasive behavior (Pang et al., 2023).

Microbiome-associated inflammatory conditions enhance TGF-β signaling, which activates SMAD-dependent transcriptional programs in stromal and epithelial cells, promoting extracellular matrix deposition, fibrosis, and epithelial–mesenchymal transition. Resulting changes increase tissue stiffness and reinforce invasive growth and metastatic dissemination (Chen et al., 2025a).

Microbial colonization within tumor tissues has been associated with local cytokine gradients and proteolytic activity, establishing a stromal microenvironment permissive to invasion through coordinated regulation of MMP activity, adhesion signaling, and extracellular matrix turnover (Guo and Deng, 2018; Li, 2023).

### Metabolic microenvironment

Microbiome-driven remodeling of the metabolic microenvironment reflects the cumulative effects of microbial metabolites on nutrient availability, immune-cell metabolism, hypoxia-associated signaling, and tumor-cell adaptation within tumor tissues. SCFA-mediated epigenetic regulation and bile acid receptor signaling contribute to metabolic reprogramming and immune suppression according to local metabolite concentration, tissue context, and immune-cell metabolic state. These microbiome-associated metabolic alterations influence T cell persistence, inflammatory signaling, and adaptation to nutrient-restricted tumor environments (Feng et al., 2024; Yao et al., 2026b). Butyrate-producing bacteria such as *Faecalibacterium prausnitzii* increase local SCFA concentrations, promoting histone deacetylase inhibition in CD8^+^ T cells and supporting effector function and persistence within tumor-associated immune compartments (Zhu et al., 2023).

Lactate accumulation within tumor regions signals through stabilization of hypoxia-inducible factor 1α (HIF-1α) in immune cells and tumor cells. HIF-1α activation induces transcriptional programs that suppress cytotoxic T-cell activity, increase expression of immune checkpoint molecules, and promote glycolytic metabolism, establishing an immunosuppressive and hypoxic microenvironment (Gu et al., 2025).

Bile acids generated by microbial metabolism activate FXR and TGR5 receptors, modulating metabolic and immune signaling pathways. FXR signaling regulates lipid and glucose metabolism in tumor and stromal cells, while TGR5 activation suppresses pro-inflammatory cytokine production and alters macrophage polarization, contributing to immune suppression and metabolic adaptation (Lin et al., 2025c).

Amino acid–derived microbial metabolites regulate immune cell metabolism through receptor-dependent pathways. Tryptophan catabolites activate the aryl hydrocarbon receptor (AhR), inducing transcriptional programs associated with regulatory T cell differentiation and reduced effector T cell function, further limiting antitumor immunity (Hezaveh et al., 2022).

## Microbiome and tumor initiation

Tumor initiation involves host–microbe interactions that contribute to initial carcinogenic processes. Microbial dysbiosis disrupts epithelial homeostasis and alters epithelial–immune crosstalk, linking environmental exposures with molecular changes in host cells. Microbial communities contribute to early carcinogenesis through inflammatory signaling, genotoxic stress, epigenetic reprogramming, and modulation of oncogenic pathways (Zhang et al., 2024).

Chronic inflammation drives tumor initiation in microbiome-associated carcinogenesis. Dysbiotic microbial communities activate innate immune receptors, leading to persistent NF-κB signaling and production of pro-inflammatory cytokines such as IL-6 and TNF-α. Sustained cytokine exposure promotes epithelial proliferation, reduces apoptosis, and increases cellular turnover, conditions associated with mutation accumulation. Loss of epithelial barrier integrity enables microbial translocation and sustained immune activation. Experimental evidence linking *Streptococcus anginosus* to inflammation-associated tumor initiation is currently strongest in gastric cancer models. Persistent colonization promotes gastritis, parietal cell atrophy, mucinous metaplasia, and dysplastic progression, supporting a defined sequence connecting chronic bacterial colonization with early gastric carcinogenesis (Mao et al., 2025; Fu et al., 2024).

Microbial metabolism contributes to tumor initiation through generation of reactive oxygen species (ROS) and genotoxic intermediates. Reactive molecules such as hydrogen peroxide induce DNA damage and mutagenesis, including DNA oxidation, strand breaks, and mutagenic lesions. Bacterial toxins further increase genomic instability. Colibactin-producing *Escherichia coli* represents one of the most extensively validated microbiome-associated carcinogenic models in CRC. Colibactin induces DNA adduct formation, double-strand breaks, and mutational signatures identified in human colorectal tumors, supporting a direct association between bacterial genotoxicity and colorectal carcinogenesis. Additional microbial metabolites, including secondary bile acids and acetaldehyde-derived compounds, contribute to oxidative stress and genomic instability in gastrointestinal tumor models (Yao et al., 2026b; Dziubańska-Kusibab et al., 2020).

Microbial signaling also modifies host gene expression through epigenetic mechanisms. Microbiota-derived metabolites modulate DNA methylation, histone modifications, and chromatin accessibility. SCFAs regulate histone deacetylase activity and alter transcriptional programs controlling immune responses and epithelial integrity. Microbial-derived signals influence noncoding RNA expression and transcriptional regulation through interactions between host epigenetic machinery and microbial metabolites. Bidirectional regulation between microbiota and host epigenome supports stable transcriptional changes that favor early neoplastic transformation (Pepke et al., 2024; Pérez Escriva et al., 2025).

Disruption of epithelial barrier function enables microbial translocation and sustained immune activation. Increased permeability allows luminal microbes and microbial products to access epithelial and subepithelial compartments, exposing host tissues to LPS and other microbial ligands that maintain receptor-dependent signaling. Barrier breakdown also facilitates microbial colonization of damaged epithelial niches and persistence within mucosal environments. In gastric cancer models, *Streptococcus anginosus* adheres to epithelial cells through Annexin A2-mediated interactions, disrupts mucosal barrier integrity, and activates MAPK signaling pathways associated with increased proliferation and reduced apoptosis. These findings support a tumor-specific mechanism linking microbial colonization with epithelial remodeling and dysplastic progression in gastric tissues (Fu et al., 2024; Zhang et al., 2025c; Yao et al., 2026b).

Microbial components and metabolites interact with host receptors and activate intracellular signaling pathways, including MAPK, NF-κB, and Wnt/β-catenin. Binding of bacterial surface proteins to epithelial receptors activates MAPK signaling, promoting cell proliferation and reducing apoptosis. Microbial metabolites, including bile acids, are linked to modulation of Wnt/β-catenin signaling and inflammatory pathways, supporting altered cell cycle regulation and maintenance of pro-tumorigenic transcriptional states (Wu et al., 2026; Zhao et al., 2023).

## Microbiome and tumor progression

Microbial communities have been associated with tumor progression through modulation of oncogenic signaling, immune suppression, vascular remodeling, and metastatic competence. Tumor-associated microbiota activate pathways such as Wnt/β-catenin and PI3K-AKT, supporting tumor cell survival, proliferation, and resistance to apoptosis. Bacterial interactions with host receptors, including integrins and E-cadherin, initiate downstream signaling that enhances cell-cycle progression. Tissue-resident microbiota correlate with hypoxia, metabolic alterations, and adverse prognosis, linking proliferative signaling to broader microenvironmental remodeling (Zhang et al., 2025a; Ivleva and Grivennikov, 2022; Shi et al., 2025).

Immune suppression mediated by microbiome-associated inflammatory signaling has been most consistently characterized in CRC. Intratumoral *Fusobacterium nucleatum* activates IL17/NF-κB/RelB signaling pathways, promoting recruitment of tumor-associated neutrophils and induction of PD-L1 expression, which suppresses cytotoxic T cell activity. Similar inflammatory associations have been observed in additional gastrointestinal malignancies, although mechanistic conservation across tumor types remains incompletely established (Zhang et al., 2025b, Zhang et al., 2025c).

Microbiota have been associated with VEGF-related signaling and endothelial activation through TLR-dependent pathways, increasing vascular permeability and neovascularization. Microbial-derived metabolites and pathogen-associated signals regulate hypoxia-associated transcriptional programs, reinforcing angiogenic signaling. Resulting vascular structures support tumor growth and facilitate tumor cell dissemination (Zhang et al., 2025c; Xie et al., 2025).

Microbial communities have been associated with invasion- and metastasis-related phenotypes, including epithelial–mesenchymal transition, extracellular matrix remodeling, and immunosuppressive niche formation. However, most mechanistic evidence derives from gastrointestinal and CRC models (Islam et al., 2026). Experimental studies indicate that microbiome-associated signaling may contribute to epithelial–mesenchymal transition, extracellular matrix remodeling, and increased tumor-cell motility. Microbial induction of cytokines, chemokines, and proteolytic enzymes has also been linked to pre-metastatic niche formation and recruitment of immunosuppressive cells in distant organs. In selected experimental systems, intratumoral bacteria have been shown to co-migrate with tumor cells and increase resistance to mechanical stress during circulation, supporting a potential role in metastatic dissemination (Fu et al., 2022). Spatial and sequencing-based analyses have additionally identified microbial signals in both primary tumors and metastatic lesions, including brain metastases, suggesting preservation of tumor-associated microbial signatures across metastatic sites (Morad et al., 2025). Metastatic disease has also been associated with reduced microbial diversity and enrichment of pathogenic taxa (Xia et al., 2025).

## Microbiome and therapeutic response

Microbiome–host interactions have been associated with variability across immunotherapy, targeted therapy, and chemotherapy through effects on immune activation, metabolic signaling, and drug biotransformation. Interindividual variability in treatment outcomes reflects differences in microbial composition and function that influence antigen presentation, cytokine signaling, and systemic immune tone (Clavijo-Salomon and Trinchieri, 2025; Situ et al., 2025).

Immune checkpoint inhibitors (ICIs) efficacy may be influenced by microbiome composition and function, although the magnitude and reproducibility of these associations remain variable across cohorts and tumor types. Distinct microbial configurations are associated with response, whereas antibiotic-induced dysbiosis reduces efficacy and correlates with impaired immune activation. Functional transfer experiments demonstrate causality, as fecal microbiota transplantation (FMT) from responders restores sensitivity to PD-1 blockade in refractory patients and reproduces response phenotypes in preclinical models. Microbial regulation of dendritic cell activation and antigen presentation determines the magnitude of tumor-specific T cell priming and downstream responsiveness to checkpoint blockade (Zarour and Trinchieri, 2026; Macandog et al., 2024) (**Figure 1C**).

Microbiome-derived metabolites and microbial antigens modulate CD8^+^ T cells and antigen presentation. SCFAs, indoles, and bile acids engage host receptors to control immune cell differentiation and inflammatory signaling. Microbial peptides with structural homology to tumor-associated antigens generate cross-reactive CD8^+^ T cell responses that enhance tumor recognition during immunotherapy (Zitvogel et al., 2024). In melanoma models, microbiota-derived formate enhances cytotoxic CD8^+^ T cell activity through NRF2-dependent signaling and improves responsiveness to immune checkpoint blockade, supporting a metabolite-dependent mechanism linking microbial metabolism with antitumor immunity in specific therapeutic contexts (Phelps et al., 2025).

CD8^+^ T cell functionality represents a central determinant of therapeutic response. Microbial signals regulate cytotoxic differentiation, interferon-γ production, and tumor-specific expansion required for effective checkpoint inhibition. Disruption of microbial homeostasis reduces T cell priming, triggers T cell exhaustion, and promotes immunosuppressive networks that limit therapeutic benefit (**Figure 1D**). Gut-derived metabolites and immune cells reach tumor sites through systemic circulation and contribute to shaping local immune composition and function (Chiodelli et al., 2026).

Microbiome–drug interactions may modify drug metabolism and bioavailability through pharmacokinetic and pharmacodynamic mechanisms. Microbial genomes encode enzymatic pathways capable of transforming xenobiotics, altering drug bioavailability, and modifying host signaling responses. Pharmacologic agents reshape microbial composition, while microbial enzymes convert drugs into active, inactive, or toxic metabolites, contributing to variability in efficacy and adverse effects. Modulation of host pathways, including cytochrome P450 systems, links microbial metabolism to systemic drug exposure and therapeutic outcomes (Bolte et al., 2025; Wilson and Nicholson, 2017; Dempsey and Cui, 2019).

In hepatic tumors, bile acid metabolism represents a major interface between microbial activity and immune regulation. Accumulation of primary and secondary bile acids impairs CD8^+^ T cell function through oxidative and endoplasmic reticulum stress pathways, limiting response to PD-1 blockade. These observations highlight a liver-specific metabolic context in which microbiome-derived bile acid signaling directly shapes therapeutic responsiveness (Varanasi et al., 2025; Lin et al., 2025d; Bautista et al., 2025c).

Chemotherapy response and toxicity are modulated by microbial enzymatic activity and host–microbiome interactions. Bacterial β-glucuronidases reactivate inactive drug metabolites in the intestine, as shown for irinotecan, increasing local toxicity and altering therapeutic outcomes. Additional mechanisms include microbial modulation of drug absorption, immune-mediated enhancement of tumor cell apoptosis, and intracellular drug sequestration, contributing to variability in treatment response (Böhm et al., 2025).

Enterohepatic circulation integrates microbial metabolism with host drug processing. Microbial modification of bile acids and drug conjugates enables reactivation and recirculation of active compounds, prolonging systemic exposure and influencing both efficacy and toxicity. This process links microbial enzymatic activity with host metabolic pathways and defines an additional layer of regulation over pharmacological responses (Larabi et al., 2023; Anand and Mande, 2022).

## Microbiome-targeted therapeutic strategies

Microbiome-targeted therapeutic strategies alter immune signaling and microbial metabolism through defined effects on immune activation, microbial metabolite production, and signaling pathways that regulate tumor progression. Modulation of the gut microbiome influences antigen presentation, cytotoxic T cell activity, and immunosuppressive circuits within the TME. Current approaches include probiotics and prebiotics, FMT, dietary modulation, and selective antimicrobial strategies (**Table 2**) (Abdeen et al., 2025; Gamrath et al., 2025; Bautista et al., 2025a).

**Table 2:** Microbiome-targeted therapeutic strategies in oncology including mechanisms, clinical maturity, limitations, and representative evidence.

Strategy	Proposed mechanism	Clinical maturity	Main limitations	Reference
FMT	Restores immune-associated microbial composition and may enhance responsiveness to immune-checkpoint inhibitors through immune reprogramming and recovery of beneficial microbial taxa	Early clinical trials	Donor variability, safety concerns, lack of standardized preparation and administration protocols, limited long-term validation	(Davar et al., 2021)
Probiotics	Promote SCFA production, dendritic-cell activation, antigen presentation, and CD8^+^ T-cell responses in a strain-dependent manner	Preclinical with limited clinical evidence	Strain specificity, inconsistent colonization efficiency, limited reproducibility across patients, insufficient oncology-specific trials	(Sivan et al., 2015)
Dietary modulation	Alters microbial composition and metabolite availability, including fiber-associated enhancement of microbial diversity and immunotherapy responsiveness	Observational studies and early intervention trials	High interindividual variability, dietary adherence challenges, confounding by lifestyle and treatment-associated factors	(Spencer et al., 2021)
Selective antibiotics	Reduces pathogenic or immunosuppressive microbial taxa associated with tumor-promoting inflammation and therapeutic resistance	Experimental/preclinical	Dysbiosis risk, depletion of beneficial commensals, off-target microbial disruption, possible impairment of immunotherapy response	(Routy et al., 2018)
Engineered bacteria	Enables targeted intratumoral delivery of immunomodulatory molecules, metabolites, or therapeutic payloads within tumor-associated niches	Experimental	Biosafety concerns, regulatory barriers, delivery control, limited translational validation in humans	(Leventhal et al., 2020)

Probiotics and prebiotics increase the abundance of commensal taxa with immunomodulatory capacity. *Bifidobacterium* and *Lactobacillus* species enhance dendritic cell activation and promote CD8^+^ T cell expansion, resulting in increased antitumor immune responses. Microbial fermentation products, particularly SCFAs, regulate immune function through inhibition of histone deacetylases and subsequent effects on T cell differentiation and effector activity. Long-term intake of probiotic-containing foods is associated with reduced CRC incidence in tumor subgroups characterized by microbial infiltration, supporting a context-dependent effect linked to microbial composition and epithelial barrier function. Prebiotics sustain these effects by providing substrates for commensal metabolism, maintaining barrier integrity and limiting inflammatory signaling associated with dysbiosis (Jiang et al., 2024; Kim et al., 2024; Bautista et al., 2026a).

FMT restores microbial diversity and modifies immune-associated microbial composition within the gut ecosystem. Available clinical evidence in oncology derives predominantly from small phase I studies and non-randomized cohorts, most commonly involving melanoma patients treated with anti–PD-1 therapy. Early findings indicate that FMT from immunotherapy responders may improve antigen presentation, increase CD8^+^ T cell infiltration, and partially restore responsiveness to immune checkpoint blockade in selected refractory patients. However, interpretation remains limited by substantial heterogeneity across studies. Reported outcomes vary according to donor selection criteria, baseline recipient microbiome composition, tumor type, conditioning regimens, route of administration, and concurrent therapies (McQuade et al., 2019; Wekking et al., 2025; Routy et al., 2023).

Donor-related variability represents a major obstacle for clinical standardization. Differences in microbial diversity, functional metabolic capacity, pathogen screening protocols, and abundance of immunologically relevant taxa directly influence microbial engraftment and downstream immune effects. Regulatory heterogeneity across countries further complicates clinical implementation. In some jurisdictions, FMT is permitted under restricted clinical-use or compassionate-access programs, whereas other regulatory agencies classify FMT as a biological product requiring formal authorization, standardized manufacturing conditions, and extensive safety screening. These differences complicate multicenter reproducibility and limit broad clinical implementation in oncology (Yadegar et al., 2024; Hoffmann et al., 2025b).

Clinical observations further indicate increased tumor-infiltrating lymphocytes, interferon-associated transcriptional programs, and improved responsiveness to immune checkpoint inhibitors following FMT. Nevertheless, most available evidence remains exploratory, underpowered, and based on limited patient cohorts. Large randomized controlled trials incorporating standardized donor selection, longitudinal microbiome monitoring, and integrated immune profiling remain necessary to determine therapeutic efficacy, safety, and durability across different cancer types (Yang et al., 2023; Ramesh et al., 2026).

## Limitations and methodological challenges

Methodological limitations in microbiome-TME research arise from constraints in sequencing resolution, study design, confounding structure, and data integration, collectively restricting mechanistic interpretation and clinical translation. Widely used sequencing approaches generate method-dependent representations of microbial communities. Amplicon-based 16S rRNA sequencing enables cost-efficient profiling but captures only a subset of microbial diversity, lacks strain-level resolution, and is affected by primer bias and variable gene copy numbers. Shotgun metagenomics provides higher taxonomic resolution and enables functional characterization across multiple microbial kingdoms, but introduces greater computational demands, host DNA interference, and dependence on reference genome completeness (Tegegne and Savidge, 2025). Non-bacterial microbial compartments remain comparatively underrepresented in cancer microbiome studies. Although emerging evidence supports contributions of the virome and mycobiome to immune regulation and tumor-associated inflammation, analytical characterization of fungal and viral communities remains limited by incomplete reference databases, lower detection sensitivity, and inconsistent classification strategies across sequencing platforms (Ding et al., 2025; Matijašić et al., 2020). Comparative analyses demonstrate discordance between 16S and shotgun outputs in taxonomic assignment and diversity metrics, particularly at lower taxonomic levels, reflecting differences in sequencing depth, database curation, and analytical pipelines. The absence of standardized analytical pipelines across studies further amplifies variability and limits reproducibility of microbiome-derived signatures (Teixeira et al., 2024).

A central limitation lies in the difficulty of establishing causal relationships between microbiome alterations and tumor-associated phenotypes. Most human studies rely on observational designs, making it challenging to determine whether microbial changes precede tumorigenesis or arise as a consequence of disease progression, treatment exposure, or host metabolic alterations. Although approaches such as Mendelian randomization and mediation analyses attempt to reduce confounding and reverse causation, their validity depends on robust instrumental variables and cross-population consistency, which remain limited. Preclinical systems, including germ-free models, organoids, and ex vivo platforms, enable controlled interrogation of host-microbiome interactions, yet fail to fully reproduce human immune complexity, spatial heterogeneity, and ecological dynamics (Lin et al., 2025b; Fierer et al., 2025). Longitudinal and multi-layered study designs, as highlighted by integrative microbiome initiatives, are required to capture temporal dynamics and strengthen causal inference, but remain insufficiently implemented (Taheriyoun et al., 2026).

Another major limitation involves uneven distribution of evidence across microbiome-associated mechanisms. Many proposed host–microbiome interactions derive from observational cohort studies or preclinical systems and lack validation in prospective clinical trials. Mechanistic findings obtained in colorectal and gastrointestinal cancer models are frequently extrapolated to other malignancies despite substantial differences in tissue architecture, immune composition, microbial localization, and metabolic conditions (Ali et al., 2026).

Confounding factors limit interpretation of microbiome–tumor associations. Microbial composition is highly dynamic and influenced by antibiotics, diet, comorbidities, and therapeutic interventions, each capable of independently reshaping microbial communities and masking tumor-specific effects. Inter-individual variability driven by host genetics, environmental exposures, and lifestyle factors further limits reproducibility across cohorts and reduces the generalizability of proposed biomarkers (Zhao et al., 2026). In low-biomass environments such as tumor tissues, contamination becomes a dominant technical constraint. Minimal exogenous DNA introduced during sampling, extraction, or sequencing can exceed the endogenous microbial signal, generating false-positive taxa and misleading biological interpretations. Variability in sampling protocols, laboratory environments, and decontamination strategies further undermines cross-study comparability and emphasizes the need for rigorous contamination-aware study design (Wang et al., 2025; Bars-Cortina et al., 2024).

Host-related biological variables further contribute to microbiome heterogeneity and reduce reproducibility across cohorts. Sex influences immune regulation, hormonal signaling, and microbial composition, generating differences in microbial metabolite production and inflammatory responses within the tumor microenvironment. Aging alters microbial diversity, epithelial barrier integrity, and immune function, modifying host–microbiome interactions and therapeutic response patterns. Ethnicity, geographic background, dietary habits, and environmental exposures additionally shape microbial community structure and functional activity, producing population-dependent microbial profiles. Host genetic variation also affects microbial colonization, immune sensing pathways, and inflammatory signaling, contributing to interindividual variability in microbiome-associated tumor phenotypes. These sources of heterogeneity complicate biomarker validation and limit cross-study comparability, emphasizing the need for demographically diverse and genetically characterized cohorts in microbiome-oncology research (Han et al., 2026; Yıldırım et al., 2026; Santo et al., 2025).

Integration of multi-omics data adds substantial computational complexity. Combining metagenomics with transcriptomics, metabolomics, and proteomics enables functional characterization of host–microbiome interactions but requires analytical frameworks capable of integrating heterogeneous, high-dimensional datasets. Differences in data scale, normalization requirements, and noise structure complicate feature selection and network inference, frequently resulting in inconsistent biological interpretation. Interpretation is further limited by biological heterogeneity across experimental systems. The same microbial metabolites or signaling pathways may generate divergent immune and metabolic effects depending on tumor type, metabolite concentration, host immune composition, and spatial localization within the TME. Cross-study comparisons are therefore constrained by substantial contextual variability. Large-scale integrative efforts demonstrate that functional outputs and dynamic host–microbiome interactions show stronger associations with disease phenotypes than taxonomic composition alone, highlighting the need to move beyond descriptive profiling. However, the absence of harmonized multi-omics pipelines and standardized datasets remains a major obstacle to reproducibility and clinical translation (Chetty and Blekhman, 2024; Tegegne and Savidge, 2025).

Machine learning approaches have been increasingly applied to identify microbiome-based biomarkers and predictive models in oncology. Despite promising performance in controlled datasets, many models exhibit overfitting, limited external validation, and instability of selected microbial features across cohorts. Model performance is highly sensitive to preprocessing decisions, feature selection strategies, and cohort composition, contributing to inconsistent findings and limiting clinical applicability. Improving generalizability requires rigorous validation on independent datasets, removal of technical artifacts, and incorporation of functional and multi-omics features rather than reliance on taxonomic profiles alone (Metwaly et al., 2025; Piccinno et al., 2025; Bautista and López-Cortés, 2026a).

## Conclusions and future perspectives

Microbial activity shapes tumor phenotypes through integrated effects on immune composition, stromal organization, and metabolic constraints within the TME. Microbial-associated molecular patterns and metabolites, such as LPS, SCFAs, bile acids, and amino acid derivatives, activate receptor-dependent signaling pathways that regulate dendritic-cell activation, macrophage polarization, T cell differentiation, cytokine production, and metabolic programs within the TME (Yao et al., 2026a; Lei et al., 2025; Bautista et al., 2026c). Experimental data indicate that these interactions involve defined signaling axes, including NF-κB, cGAS–STING, and receptor-mediated pathways linked to microbial metabolites. In breast cancer models, intracellular bacteria activate cGAS–STING–IL-17B signaling and promote neutrophil-mediated immunosuppression, whereas extracellular bacterial exposure induces distinct neutrophil states associated with antitumor activity, indicating that microbial localization influences immune outcomes (Jia et al., 2025).

Detection of microbial signals in tumors requires careful interpretation. Low-biomass samples are susceptible to contamination and classification errors, which can lead to false-positive or false-negative findings. Computational approaches incorporating decontamination steps and reference-based alignment improve specificity and enable identification of tumor-associated microbial signatures linked to host molecular features, including alterations in glycosylation pathways. Independent validation remains necessary. Imaging-based methods and spatial analyses have demonstrated intracellular bacterial signals in tumor, immune, and stromal compartments and have shown regional associations with antimicrobial and immunometabolic programs in the TME (Ghaddar et al., 2026; Morad et al., 2025).

Most available human data are cross-sectional and do not resolve temporal relationships between microbial variation and tumor progression. Longitudinal designs are required to determine whether microbiome changes precede disease progression, arise during tumor evolution, or reflect treatment exposure. Repeated sampling across treatment timelines is particularly relevant in immunotherapy, where microbiome composition and modulation strategies, including FMT and dietary interventions, have been associated with differences in response and toxicity. Standardized sampling protocols and consistent clinical metadata will be necessary to reduce variability across studies (Xie et al., 2025; Yao et al., 2026b).

Current data indicate that microbial metabolites regulate antigen presentation, T cell activation, and inflammatory signaling, but causal contributions within complex tumor environments remain insufficiently resolved. Controlled experimental systems, including gnotobiotic models, organoids, and microfluidic platforms, will be required to define metabolite-specific and receptor-dependent effects with greater precision. Comparative evaluation of gut-derived and intratumoral microbial activity is also necessary to clarify spatially restricted functional interactions across tumor-associated niches (Yao et al., 2026a; Situ et al., 2025).

Mechanistic conservation across cancer types remains incompletely defined. Although inflammatory signaling, metabolite-mediated immune modulation, and microbiome-associated therapeutic effects have been identified in multiple malignancies, experimentally validated interactions derive predominantly from colorectal and gastrointestinal tumor models. Differences in epithelial structure, immune composition, tissue metabolism, and microbial localization generate tumor-specific host–microbiome interactions that restrict direct extrapolation across cancers. Comparative analyses across anatomically distinct tumor systems will be required to distinguish conserved microbiome-associated pathways from tissue-restricted mechanisms (Luo et al., 2025; Ivleva and Grivennikov, 2022; Bautista et al., 2025b).

Integration of multi-omics data is necessary to characterize microbiome function. Taxonomic profiling alone does not capture metabolic activity or host interaction. Combined metabolomic, transcriptomic, proteomic, and spatial analyses allow identification of microbial products, host receptors, and downstream pathways within the same system. Multi-omics studies have identified links between microbial composition, immune signaling, and metabolic pathways associated with tumor progression and therapeutic response. Systematic integration across datasets will help define reproducible molecular patterns and identify targets for intervention (Su et al., 2025; Dong et al., 2025).

Clinical application remains limited. Microbiome-associated signatures correlate with diagnostic, prognostic, and treatment-response parameters, yet no microbiome-based biomarkers are implemented in routine clinical workflows. Translation is constrained by variability in sampling procedures, lack of standardized analytical pipelines, and limited validation across independent and clinically heterogeneous cohorts (Rodriguez et al., 2025; Li et al., 2024). Implementation will require validated assays, clear definitions of clinical use, and reproducible performance across populations. Advances in microbial detection and classification may facilitate biomarker development when combined with standardized workflows (Zhao et al., 2025).

Microbiome-targeted interventions have shown effects on immune regulation and treatment response in preclinical and early clinical settings. Approaches include FMT, probiotics, dietary modulation, and engineered microbial systems. These strategies can alter immune cell infiltration, cytokine signaling, and response to immune checkpoint inhibitors, but clinical outcomes remain variable and context-dependent. Differences in host factors, tumor type, treatment regimen, and baseline microbiome composition contribute to this variability. Controlled trials and mechanistic studies are required to define which patients benefit from specific interventions (Hajjar et al., 2026; Kim et al., 2025; Bautista et al., 2026b).

A major unresolved challenge involves distinguishing context-dependent microbiome effects from conserved tumor-promoting mechanisms. Several microbial metabolites and inflammatory pathways demonstrate opposing biological effects across tumor types and experimental systems, limiting generalized interpretation. Future studies must therefore define concentration thresholds, spatial distribution, receptor specificity, and temporal dynamics that determine whether microbiome-associated signaling promotes immune activation or immune suppression within distinct tumor ecosystems (Chen et al., 2026).

Progress in the field will depend on coordinated advances in study design, experimental validation, and analytical standardization. Longitudinal datasets, controlled model systems, and integrated multi-omics analyses will be necessary to define causal relationships and functional mechanisms. Standardized methodologies for microbial detection and biomarker validation will be required for clinical implementation. Under these conditions, microbiome-informed strategies may eventually support patient stratification and support microbiome-guided therapy in oncology (Sherwani et al., 2025).
